# The methodological quality of three foundational law enforcement drug influence evaluation validation studies

**DOI:** 10.1186/1477-5751-12-16

**Published:** 2013-11-04

**Authors:** Greg Kane

**Affiliations:** 1 10785 East Crestline Place, Englewood CO 80111, USA

**Keywords:** MESH terms, Automobile driver examination, Research design, Sensitivity and specificity, Non-MESH, Drug influence evaluation, Drug recognition expert, Drug evaluation and classification program, Sobriety testing

## Abstract

**Background:**

A Drug Influence Evaluation (DIE) is a formal assessment of an impaired driving suspect, performed by a trained law enforcement officer who uses circumstantial facts, questioning, searching, and a physical exam to form an unstandardized opinion as to whether a suspect’s driving was impaired by drugs. This paper first identifies the scientific studies commonly cited in American criminal trials as evidence of DIE accuracy, and second, uses the QUADAS tool to investigate whether the methodologies used by these studies allow them to correctly quantify the diagnostic accuracy of the DIEs currently administered by US law enforcement.

**Results:**

Three studies were selected for analysis. For each study, the QUADAS tool identified biases that distorted reported accuracies. The studies were subject to spectrum bias, selection bias, misclassification bias, verification bias, differential verification bias, incorporation bias, and review bias. The studies quantified DIE performance with prevalence-dependent accuracy statistics that are internally but not externally valid.

**Conclusion:**

The accuracies reported by these studies do not quantify the accuracy of the DIE process now used by US law enforcement. These studies do not validate current DIE practice.

## Background

### Law enforcement drug assessment

**Figure 1:**
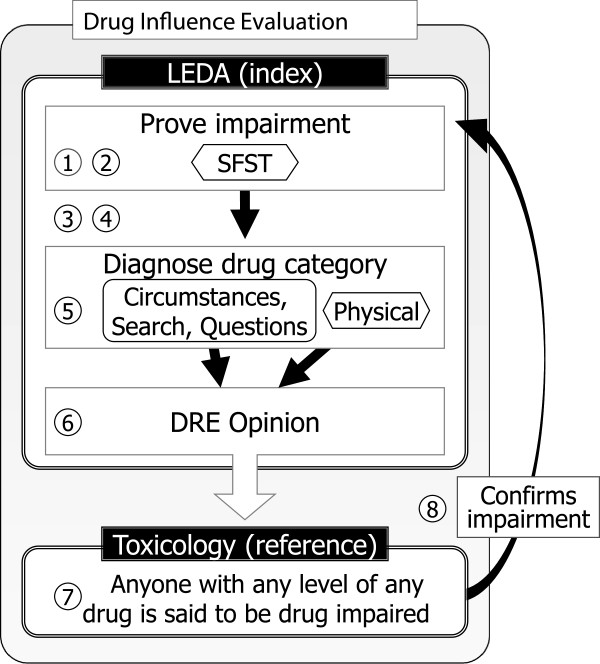
**US law enforcement Drug Influence Evaluation theory.** 1 Driver fails a Standardized Field Sobriety Test (SFST). 2 A failed SFST always proves impairment. 3 Breath alcohol test finds a low alcohol level, ruling out alcohol as the cause of impairment proven by SFST. 4 Medical evaluation by the DRE officer rules out a medical cause of impairment proven by the SFST. 5 DRE exam involving circumstantial facts, questioning, a physical search (if done), and a physical exam, is used to identify the presence in the subject’s body of a drug belonging to one of several categories. 6 A trained Drug Recognition Expert opines that the driver is, or is not, impaired by a drug belonging to a particular category. 7 Blood or urine is tested for drugs. 8 Toxicology revealing a drug in the predicted category circles back to confirm the SFST’s proof of impairment. The person must have been impaired—*How else could the officer have made this very specific drug category prediction?* Alternatively, when a drug in the predicted category is not found, some validation study accuracy calculations consider any drug in any other category as proving impairment—*After all the officer predicted a drug, and a drug was found.*

A Drug Influence Evaluation (DIE) is a formal assessment of an impaired driving suspect, performed by a law enforcement officer called a Drug Recognition Expert (DRE). The assessment culminates with the officer’s opinion that the person is, or is not, impaired by a drug belonging to one of several categories, or multiple drugs belonging to multiple categories. In criminal trials DRE officers’ opinions are used, with or without blood or urine drug toxicology testing, as evidence of drug impaired driving. When an officer’s drug category prediction matches later toxicology results, the correctness of the drug category prediction is said to confirm the officer’s opinion that the suspect was impaired by that drug, according to the theory outlined in Figure 1. Current US law enforcement DIE practice is overseen by the International Association of Chiefs of Police (IACP), which coordinates the International Drug Evaluation and Classification Program, with support from the National Highway Traffic Safety Administration (NHTSA).

In current forensic practice “Drug Influence Evaluation” usually refers to the combination of the DRE officer’s assessment of the suspect and toxicology testing. This differs from the foundational research, which calculated the accuracy with which officers’ opinions matched toxicology results, and which therefore needed technical terminology for the suspect assessment and officer’s opinion exclusive of the toxicology. Different studies used various infelicitous terms. This paper will refer to the Law Enforcement Drug Assessment (LEDA) as consisting of two parts 1) the Suspect Assessment (SA), in which data is gathered, and 2) the officer’s DRE Opinion as to whether or not the suspect’s driving was impaired by drugs. Toxicology testing may follow, but is not part of a LEDA as the term is used here. The DIE validation studies investigated in this paper calculated the accuracy with which LEDA SAs led to DRE Opinions that correctly predicted toxicology results.

The idea of a formal LEDA was developed by Los Angeles police officers in the 1970s, and used in local criminal prosecutions without published diagnostic accuracy research. In the early 1980s the National Highway Traffic Safety Administration (NHTSA) adapted the Los Angeles program for national use [1]. NHTSA directly sponsored two police drug recognition validation projects, identified here as Bigelow [2] and Compton [3], and through the Arizona Department of Transportation funded a third, Adler [4]. These three police drug recognition validation projects were printed by NHTSA (Bigelow, Compton) and the Arizona Department of Transportation (Adler). Peer reviewed studies of LEDA-like testing have also been reported [5,6] including papers investigating refined interpretation techniques [7-9] or focused on specific drugs [10-15].

### Accuracy of diagnostic tests

In general, studies of diagnostic test accuracy reveal a number of accuracy statistics, including the accuracy of the test when its answer is Yes (positive predictive value, PPV), when the answer is correct (overall accuracy), when administered to people with the target condition (sensitivity), and when administered to people without the condition (specificity). These accuracy numbers are generally all different from each other [16].

The purpose of a DIE is not simply to identify drug impairment; to be useful the DIE process must tell the difference between people who are drug impaired and people who are sober. The accuracy with which a diagnostic test identifies that difference is generally expressed with the paired statistics sensitivity and specificity [17-21], or with likelihood ratio [22-24], or the area under the ROC curve [25,26].

Some diagnostic test accuracy statistics are externally valid (generalizable to populations other than the one in the study), some are not. PPV—“arrest accuracy”, or “accuracy of the arresting officers’ toxicology predictions”—is not externally valid. It depends on prevalence [27-30]. Because the prevalence of drug impairment changes as time and location change, even when officers make exactly the same measurements and interpret them with exactly the same criteria, the accuracy of the officers’ predictions changes depending on where and when the test is administered.

### Diagnostic research methodology

Research to discover the accuracy of a diagnostic test is conceptually easy: administer the test to a group of people and see if it works. The test being tested is the “index test”. Results of the index test are compared with results of a gold standard “reference test”. The research question is, “How accurately do index test results predict the (true, gold standard) reference test results?”

Research to discover the accuracy of diagnostic tests is in practice very challenging. Devils lurk in the details. What would seem to be small variations in research methodology lead to large systematic errors—biases—in a test’s apparent accuracy [31-33]. Bias affects the validity of a study’s findings [34-36]. It is important not to take reported diagnostic test accuracies at face value, but instead to evaluate the underlying research for methodologies known to cause bias [37-39]. Various authors and groups, particularly those interested in meta-analyses, have suggested checklists and scoring methods to do this in a systematic way [40-43]. Details differ, the methodologies targeted overlap.

The Quality Assessment of Diagnostic Accuracy Studies (QUADAS) tool is a set of fourteen questions that investigate the methodologic quality of scientific studies that quantify diagnostic test performance [40]. The questions identify research methodologies known to bias the accuracies research discovers. QUADAS identifies individual methodologic shortcomings, it does not provide a meaningful composite quality score [44]. Empirical evidence shows that methodologic shortcomings may cause studies to overestimate the accuracy of diagnostic tests [45]. When reviewers use the tool, their judgments about methodologic quality show high rates of agreement [46-48]. QUADAS use is increasing [49].

This paper uses the QUADAS tool to investigate the methodological quality of the scientific research commonly offered in American courts as evidence of the validity and reliability of the US law enforcement DIE process.

## Results and discussion

### Study selection

The IACP’s The International Standards of the Drug Evaluation and Classification Program [50], hereafter Standards identifies DRE training as having been “validated through both laboratory and field studies conducted by Johns Hopkins University”. This cannot be correct. The Johns Hopkins DRE lab study was Bigelow; there was no Johns Hopkins DRE field study. At the IACP, the Executive director did not respond. The two IACP TAP officials did respond. Neither identified any DIE testing standards. The TAP’s medical expert did give assurances that the DIE process includes “guidelines” with a scientific basis, but he did not name specific validating research. Pressed for specifics, both TAP officials quit answering emails or certified mail. The DECP.org web site states that “NHTSA, and various other agencies and research groups examined the DEC program. These various studies demonstrated that a properly trained DRE can successfully identify drug impairment”. [http://www.decp.org/about, retrieved March 4, 2013] but the studies themselves are not named. The DRE Student Manual identifies the accuracy of current DIE practice as being demonstrated by Bigelow, Compton and Adler. The NCDD legal expert identified two studies—Bigelow and Compton—as the those frequently cited in American criminal trials as evidence of DIE accuracy, and a third study—Adler—as being important because it is identified as evidence of the accuracy of DIEs in the official DRE Student Manual. He identified no other studies as pertinent to this paper’s defining question. The studies selected for analysis were Bigelow, Compton and Adler.

### Reported accuracies

Bigelow, Compton and Adler each reported a number of diagnostic accuracy related statistics, some of which were highlighted:

Bigelow: “Of subjects judged to be intoxicated the correct drug class was identified on 91.7% of occasions. Overall, in 98.7% of instances of judged intoxication the subject had received some active drug” [Bigelow, page 16].

Compton: “When the DREs claimed drugs other than alcohol were present they were almost always detected in the blood (94% of the time)” [Compton, page 22].

Adler: “DRE decisions were supported by laboratory analysis for 404 (83.5%) of the 484 specimens” [Adler, page 33].

### QUADAS Items

The studies’ research methodologies are summarized in Appendix 1. QUADAS questions and answers are reported in Table 1. The QUADAS tool identified research methodologies expected to cause bias and to make the accuracies the studies reported not internally or externally valid. These are discussed below and summarized in Appendix 2.

**Table 1:** QUADAS results

	**Bigelow**	**Compton**	**Adler**
1. Was the spectrum of patients representative of the patients who will receive the test in practice?	No	No	No
2. Were selection criteria clearly described?	No	No	No
3. Is the reference standard likely to correctly classify the target condition?	No	No	No
4. Is the time period between reference standard and index test short enough to be reasonably sure that the target condition did not change between the two tests?	Yes	Yes	Yes
5. Did the whole sample or a random selection of the sample, receive verification using a reference standard of diagnosis?	Yes	No	No
6. Did patients receive the same reference standard regardless of the index test result?	Yes	No	No
7. Was the reference standard independent of the index test (i.e. the index test did not form part of the reference standard)?	No	No	No
8. Was the execution of the index test described in sufficient detail to permit replication of the test?	No	No	No
9. Was the execution of the reference standard described in sufficient detail to permit its replication?	Yes	No	No
10. Were the index test results interpreted without knowledge of the results of the reference standard?	No	No	No
11. Were the reference standard results interpreted without knowledge of the results of the index test?	Yes	No	No
12. Were the same clinical data available when test results were interpreted as would be available when the test is used in practice?	n/a	n/a	n/a
13. Were uninterpretable/ intermediate test results reported?	No	No	No
14. Were withdrawals from the study explained?	No	No	No

#### QUADAS Item 1—spectrum bias, forensic spectrum bias

It is generally easier to diagnose severe disease than mild disease. Studies with sample populations skewed toward severe disease discover inflated sensitivities that do not reflect the accuracy of the identical test in other settings. When a study selects subjects in a way that distorts the spectrum of disease severity, a systematic error arises called *spectrum bias*.

Bigelow’s lab study confirmed that LEDA accuracy is subject to spectrum bias. When subjects were given the same drugs at different doses, DRE Opinions were less accurate at spotting people given the lower dose than they were at spotting people given the higher dose. This effect was found for every drug tested this way. Bigelow highlighted LEDA accuracies derived from subjects dosed at high doses, three and six times the usual prescription level. Bigelow’s accuracies were subject to spectrum bias.

Compton and Adler’s study groups were distorted by the inclusion only of people arrested for chemically impaired driving. These studies were both subject to what may be called *forensic spectrum bias*—spectrum bias arising in sample populations preselected to be guilty beyond a reasonable doubt.

Compton’s study sample was made up of people who had already been identified as chemically impaired and arrested by non-DRE officers. Drivers were then screened again and included in the study only if their impairment was apparent on a “cursory exam” [Compton, page 34]. Seventy-two percent of Compton’s subjects had multiple drugs on board. The accuracies Compton reported were the accuracies of the LEDA applied to people whose impairment was so obvious it was already apparent to non-DRE experts and judged to be provable beyond a reasonable doubt *before* the LEDA SA physical exam was done. Compton’s accuracies were subject to forensic spectrum bias.

Adler likewise reports the accuracy of the LEDA on people who were arrested—people against whom experienced officers concluded a beyond-a-reasonable-doubt legal case could be made. Most had multiple drugs on board. Adler’s accuracies were subject to forensic spectrum bias.

#### QUADAS Item 2—selection bias

When research selects subjects in a way that distorts test-result-relevant characteristics of the sample of people in the study compared to the population of people to whom the test will be administered later, a systematic error arises called *selection bias*.

Bigelow’s study group was distorted by the selection only of healthy young men prescreened to pass the SFST and then further trained to do the SFST. These subjects will have been more likely to pass the SFST than would the older, the unhealthy, the uncoordinated people, and the people not screened to pass the SFST who are part of the population the SFST/ LEDA is applied to in police practice outside the lab. The accuracy of the SFST administered to Bigelow’s sample will be different from the accuracy of the SFST (and thus the LEDA) administered to the population in general. Bigelow’s accuracies were subject to selection bias.

Compton and Adler’s study groups were distorted by the fact that neither study enrolled a series of consecutive drivers stopped by police. Some drivers were stopped, assessed and not given a LEDA; they were excluded from the studies’ accuracy calculations. Other drivers were stopped, assessed and did have a LEDA administered; the studies’ reported accuracies depend on the performance of just this group. The accuracies Compton and Adler discovered will have depended on how officers made these administer/ don’t administer decisions—on how they selected subjects. These selection decisions will have been made in accordance with local police policies, local prosecutors’ policies, local demographics, local drug use patterns, and each officers’ unstandardized personal practices. The accuracies reported by Compton and Adler were subject to selection bias.

#### QUADAS Item 3—misclassification bias

Studies of diagnostic test accuracy quantify the ability of an index test to predict the results of a reference test. The reference test is assumed to be a gold standard that correctly identifies the target condition with 100% accuracy. If the reference test fails to diagnose the target condition correctly, a systematic error arises called *misclassification bias*.

In drugged driving prosecutions the LEDA’s target condition is the subject’s driving, but none of the three studies used driving performance or physical or mental impairment as their reference test. Bigelow’s reference test was the fact a subject was given a medicine. Compton and Adler’s reference test was blood or urine testing for drugs. In each study the reference test and the target condition were different.

The question raised by QUADAS Item 3 is, “Does drug in the blood or urine correctly classify people as drug impaired?” The answer is, it may not. Low levels of drugs and metabolites are found in the body hours or even, depending on the drug, days after impairing effects have ended. The studies themselves recognized they were subject to misclassification bias. Compton noted,

“There is no way to determine objectively whether the suspects were actually too ‘impaired’ to drive safely. The fact that drugs were found in a suspect’s blood does not necessarily mean the suspect was too impaired to drive safely” [Compton, page 15].

Each study sought to cure it’s misclassification bias with the theory outlined in Figure 1: using a physical examination tuned to specific drugs, officers made amazingly specific toxicology predictions. An officer’s correct prediction of cocaine could not have happened unless the subject really was impaired by the cocaine later found by toxicology. Adler wrote:

“Because a specimen may test positive at a time when the suspect is not under the influence of marijuana, a DRE evaluation is crucial. Importantly, unless a marijuana positive from the laboratory is corroborated with evidence of impairment at the time of the evaluation, it does not speak to the question of drug influence” [Adler, page 40].

This theory has a number of problems. First, it is circular. The purpose of a DIE validation study is to discover whether LEDAs do in fact accurately identify drug impairment. That proof may not derive from its own conclusion.

Second, the theory conflates side effects with impairment. The mere fact the presence of a drug may be identified by stereotypic physical side effects need not indicate the drug is causing mental impairment.

Third, the theory depends on study officers having made their predictions based on a physical exam. But officers had access to other facts—circumstances, answers to questions, searches. It may be officers used these other facts to identify the presence of a drug in the subject’s body, and used the presence of a drug to predict the presence of a drug, the reference standard. One technique available was to ask, “Have you taken any drugs?” Adler reported 434 confessions and 682 drug predictions. If officers predicted a drug each time a driver confessed, 64% of drug predictions were made after drivers confessed. When confessions and searches that found drugs are considered together, as many as 80% of drug predictions were made in cases involving either a confession or a positive search. Because certainly some drivers both confessed and had drugs found, this is an upper bound.

If DIE validation study officers based their toxicology predictions on confessions of prescriptions from a doctor, or drug use hours ago, or searches that turned up drugs or paraphernalia, or physical signs of chronic drug use like “needle marks, skin rashes, perforation of the nasal septum” [Compton, page 5], or witness statements, or a history of drug convictions, then officers based their correct toxicology predictions on facts independent of ongoing mental impairment. To the extent this is what happened, the accuracies reported by Compton and Adler were subject to misclassification bias and the circular question DIE validation studies rely on to confirm driver impairment, “How else could officers have made specific drug predictions?” has a straightforward answer, “By selecting for inclusion in the study people who confessed or had drugs found in a search”.

Fourth, the theory depends on the false idea that validation study DRE Opinions were generally correct. They were not. The LEDA accuracies highlighted by the validation studies overstate how often DRE officers predicted drug categories correctly. Compton’s 94% accuracy counts mistaken—wrong—drug category predictions as correct so long as toxicology found literally *any* proscribed drug other than alcohol. If an officer predicted a CNS stimulant but toxicology found a CNS depressant, that wrong prediction would contribute a correct score to Compton’s 94% accuracy. Here the DIE validation studies’ misclassification bias theory asks us to believe that because the DRE officer found what he thought were signs of a stimulant that was not present, the driver must have been impaired by a depressant whose physical signs were not found. More likely wrong predictions were just that, wrong predictions, and Compton’s highlighted 94% accuracy was subject to misclassification bias.

Similarly, Adler’s highlighted 83.5% “supported by laboratory analysis” statistic removed wrong predictions from the accuracy calculation [Adler, page 33]. In fact officers correctly predicted toxicology findings in only 43% of subjects [Adler, page 34]. Adler’s highlighted accuracy was subject to misclassification bias.

Fifth and finally, the DIE validation studies’ misclassification bias theory depends on the premise that validation studies’ positive toxicology results reflected drugs present at impairing levels. Some did not. Bigelow and Adler acknowledged the problem but their methodologies did not allow it to be quantified. Compton reported that “In approximately one quarter of the cases that marijuana was detected, the blood level was reported as <1.0 ng/ml (an extremely small amount)” [Compton, page 14]. Here DIE validation studies’ misclassification bias theory is correct only if marijuana present at these levels was impairing. More likely drivers were not impaired by drugs present at extremely low levels and Compton’s reported accuracy was inflated by misclassification bias.

#### QUADAS Item 3—misclassification bias, alcohol

Neither Compton nor Adler excluded subjects whose breath alcohol concentration (BrAC) tests proved they had alcohol on board. Both studies’ accuracy calculations included drivers with alcohol levels above the current per se legal limits of 0.05 and 0.08% mg/dl. In Compton 53% of drivers in the sample group had alcohol in their system, with an average BrAC of 0.06% mg/dl, and a maximum of 0.18% mg/dl [Compton, page 14]. In Adler 19% had BrACs greater than 0.05% mg/dl, with a maximum greater than 0.20% mg/dl [Adler, page 54]. In both Compton and Adler, some subjects classified as impaired by drugs other than alcohol were certainly impaired by alcohol. The classification of drivers impaired by alcohol as impaired by drugs will have caused Compton and Adler’s reported accuracies to be distorted by misclassification bias.

#### QUADAS Item 3—misclassification bias, drug conflation

Bigelow, Compton and Adler each highlighted the accuracy with which DRE Opinions predicted “drugs”, as if “drug” were a homogenous medical entity. This methodology fails to classify LEDA results correctly.

First, “drug” is not a homogenous medical entity and the accuracy of DRE Opinions varies from drug to drug. Using Compton’s sample group as an example, sensitivity for PCP (phencyclidine) was 91%, but for cocaine 19% [Compton, page 46]. Counting a correct PCP prediction as indicating the accuracy of cocaine predictions misclassifies that PCP result and misstates the accuracy of the LEDA when an officer predicts cocaine.

Second, because “drug” is not a homogenous medical entity, bias also arises in the attribution of research accuracies to drugs and drug categories that were not studied at all. Many of the drugs now claimed for the DIE were not investigated at all by Bigelow, Compton or Adler [Table 2]. Counting correct PCP predictions (which were studied) as indicating the accuracy of dextromethorphan predictions (which were not) misclassifies study results and misstates the accuracy of the LEDA when an officer predicts dextromethorphan.

**Table 2:** DIE drug category lists

**NHTSA’s DRE student manual**	**Bigelow**	**Compton**	**Adler**
1 CNS Depressants	1 d-Amphetamine = CNS Stimulant	[1 Amphetamines (no subjects with amphetamines were tested.)]	1 Stimulants
			2 PCP
2 CNS Stimulants			3 Hallucinogen
	2 Diazepam & Secobarbital = CNS Depressant		4 Cannabis
3 Hallucinogens		2 Barbiturates	5 Inhalants
		3 Cocaine	5 Depressants
4 Dissociative Anesthetics	3 Marijuana	4 Cannabis	6 Narcotics
			7 Other
		5 Opiates	1 PCP
5 Narcotic Analgesics	[4 Narcotics (no subjects with narcotics were tested)]	6 PCP	
			2 Morphine
6 Inhalants		7 Benzodiazepines	3 Cocaine
		8 Alcohol	4 Marijuana
7 Cannabis			5 Barbiturate
			6 Benzodiazepine
			7 Methamphetamine/Amphetamine

As reflected by NHTSA’s DRE Student Manual, the list of DIE drug categories used in US law enforcement DIE practice differs from the lists validated by Bigelow, Compton and Adler, whose lists differ from each other. Adler used two different lists. CNS = Central Nervous System. DRE Student Manual, page 2–3; Bigelow, page 9; Compton, page 7; Adler pages 26, 47.

#### QUADAS Item 4—disease progression bias

Medical research may involve subjects whose reference test was performed months after the index test, during which time the medical condition progressed and became easier to identify, leading to a systematic error called *disease progression bias*.

In DIE validation studies a delay between the LEDA SA and blood or urine sampling may allow the body’s metabolism to lower drug levels below the reference test cutoff level, leading to a similar bias. Bigelow did not do chemical tests. Compton’s index and reference testing were contemporaneous. Adler considered all detectable drugs to represent impairment, regardless of how low the drug level was. Drug levels that in the less than two hour interval between arrest and sample collection fell from above to below the laboratory detection level must have been present at low levels at the time of arrest and would therefore be correctly classified as non-impairing in either case. None of the three studies are subject to disease progression bias.

#### QUADAS Item 5—verification bias

When the decision whether or not to administer the reference test depends on the outcome of the index test a systematic error arises called *verification bias.* This error distorts the arithmetic of accuracy calculations in a way that falsely increases the apparent sensitivity and decreases the apparent specificity a study discovers [51]. The errors may be substantial.

Compton and Adler administered the reference test (toxicology) only to people who failed the index test (LEDA). The sensitivities and specificities discovered by these studies are subject to verification bias (but see §Accuracies below).

#### QUADAS Item 6—differential verification bias

When more than one reference test is used and one is more accurate than the other, and when the decision about which reference test will be administered depends on the outcome of the index test, a systematic error arises called *differential verification bias*.

Bigelow is not subject to this bias. Compton administered blood tests for methaqualone and mescaline (reference) only when officers predicted those drugs (index). Adler administered a variety of drug-specific toxicology tests (reference) only when officers predicted those specific drugs (index). The accuracies reported by Compton and Alder are subject to differential verification bias.

#### QUADAS Item 7—incorporation bias

When the index test result necessarily contributes to—is incorporated into—the interpretation of reference test, a systematic error arises called *incorporation bias*. Consider a study investigating whether a hand x-ray can predict whether a finger fracture will require surgery. The study’s index test would be the hand x-ray. The reference test would be whether the patient had surgery. But in deciding whether to do surgery, the orthopedists would consider MRI scans, clinical history, physical exam—and the hand x-rays. Thus the results of the index test would contribute to the results of the reference test. This sort of incorporation leads to a bias that causes studies to discover higher apparent accuracies.

Bigelow, Compton and Adler are subject to incorporation bias in their diagnosis of impairment. As illustrated in Figure 1, LEDAs rely on the SFST to diagnose impairment. Neither Bigelow, Compton or Adler provide or consider a false positive rate for the SFST’s diagnosis of impairment. In all three studies, the SFST serves as both index test and reference test for impairment. The accuracies reported by these studies are subject to incorporation bias.

#### QUADAS Item 8—index test reproducibility

The accuracy of a diagnostic test depends on which measurements are made and how they are interpreted. Tests that make different measurements or use different interpretations will have different accuracies. The accuracies reported by Bigelow, Compton and Adler describe the accuracy of current law enforcement DIEs only to the extent current LEDA methods reproduce the LEDAs investigated in those three studies. QUADAS Item 8 asks, “Do the studies describe their LEDAs in a way that allows their index testing—and accuracies—to be reproduced?” The answer is, “No”. Bigelow, Compton and Adler did not describe the LEDAs they investigated in enough detail for that testing to be reproduced.

The LEDA SA includes the gathering of circumstantial facts, suspect questioning, and a search of the suspect. These elements of the LEDA are not standardized. None of the three studies identified which circumstantial facts officers recorded, what questions they asked, or what they searched. These elements of the validation studies’ LEDA SAs cannot be reproduced.

The LEDA SA includes a physical exam looking for “indicators” of impairment or drugs. The three studies did name some physical maneuvers officers did, with some maneuvers (e.g. pulse and blood pressure) being reproducible, and others (e.g. “eyes glassy”, “attitude”, “condition of the tongue”) being identifiable but not reproducible. None of the studies reported a standardized list of physical exam indicators. The studies’ LEDA SA physical exams are not reproducible.

The LEDA DRE Opinion derives from consideration of the LEDA SA findings. None of the three studies described what their LEDA SA findings were, or how those findings were combined to formulate DRE Opinions. The validation studies’ LEDA DRE Opinions cannot be reproduced.

Current US law enforcement DIE methods do not reproduce the LEDA methods investigated in Bigelow, Compton and Adler. The accuracies reported in these studies do not reflect the accuracy of current DIEs.

#### QUADAS Item 9—reference test reproducibility

The accuracy with which the results of an index test predict the results of a reference test may be affected by variations in how the reference test is performed. Bigelow’s reference “test”, the record of whether the subject was given a drug, is reproducible. Compton and Adler do not describe their reference tests in reproducible detail.

#### QUADAS Items 10 and 11—review bias

When diagnostic tests whose interpretation is subjective are investigated in studies that are not blinded, knowledge of one test result (reference or index) may influence the interpretation of the other test in a way that tends to make the results agree. When this happens a systematic error arises called *review bias*. When the reference test result is known at the time the index test is interpreted, the error is *test review bias*. When the index result is known at the time the reference is interpreted, the error is *diagnostic review bias*.

Bigelow’s blinding was partial. When the LEDA-like SA (index) was interpreted, officers had been told subjects would have drugs on board and what categories those drugs would be (reference). Officers were more likely to correctly choose the index DRE Opinion “CNS stimulant” because they knew “LSD” and “PCP” and “paint thinner” and “dextromethorphan” and “hypoglycemia” and “not naturally coordinated” and “alcohol” were not correct. The LEDA accuracies Bigelow reported were subject to test review bias.

Compton and Adler’s officers interpreted LEDA SAs (index) before toxicology results (reference) were available. DRE Opinions were not subject to review bias.

The studies themselves were different. Both studies reported what golfers would call Mulligan accuracies, statistics that counted LEDA DRE Opinions (index) when toxicology results (reference) showed the opinions were correct, but didn’t count DRE Opinions when toxicology showed they were wrong. For example Adler reported subjects for whom the DRE Opinion correctly predicted one drug (one true positive prediction), wrongly predicted a second (one false positive prediction) and wrongly missed a third (one false negative prediction) [Adler, page 34]. That’s one correct and two wrong index results. But Adler’s “decisions supported by laboratory analysis” accuracy calculation tallied this encounter not as one correct and two wrong predictions, but as one correct prediction. The two wrong DRE Opinions disappeared from the calculation. Compton calculated similarly.

Compton’s report that DRE Opinions “identified one or more drugs correctly in 87% of the suspects” [Compton, page 16], and Alder’s “DRE decisions were supported by laboratory analysis for 404 (83.5%) of the 484 specimens” [Adler, page 33], both relied on this methodology. Both accuracies reflect review bias. Neither number correctly quantifies the probability relevant to the US law enforcement use of DIEs, the probability that an individual LEDA DRE Opinion is correct.

#### QUADAS Item 12—clinical review bias

In studies of diagnostic tests whose interpretation is subjective, knowledge of patient characteristics like age, gender, symptoms or medical history may influence the accuracy of test interpretations. When this happens a systematic error arises called *clinical review bias*.

Because clinical facts (circumstances, questioning, searches) are defined as part of the LEDA, the accuracies reported by Bigelow, Compton and Adler cannot, by definition, be subject to this bias.

#### QUADAS Items 13 and 14—uninterpretable/ intermediate test results and study withdrawals

Generally some index test results will be uninterpretable. When uninterpretability is related to disease status, accuracy may be biased. Similarly, subjects may be withdrawn from studies for nonrandom, biasing reasons. Neither Bigelow, Compton and Adler report uninterpretable or intermediate test results or study withdrawals. Whether bias arises due to these factors is unclear.

### Accuracies

Diagnostic accuracy research is helpful when it quantifies test performance with externally valid statistics that allow later users to calculate the predictive values of the tests they administer. Bigelow, Compton and Adler reported a number of accuracy statistics, from which Bigelow and Compton highlighted some form of LEDA DRE Opinion accuracy equivalent to positive predictive value. Adler highlighted a “decisions supported by toxicology” accuracy, equivalent to overall accuracy. Adler’s statistic does not quantify the probability relevant to drugged driving prosecutions, the probability that a DRE Opinion, of “Yes, drug present” opinion was correct.

Both overall accuracy and PPV are prevalence dependent. Neither is externally valid. The LEDA accuracies reported by Bigelow, Compton and Adler are not valid for—do not quantify the accuracy of—LEDAs now administered by US law enforcement.

Compton illustrates how methodologies combining selection bias with prevalence dependent statistics may overstate the diagnostic power research discovers. Compton reported that “When the DREs claimed drugs other than alcohol were present they were almost always detected in the blood (94% of the time)” [Compton, page 22]. A simple reading would suggest this high accuracy was a product of the LEDA testing study officers did. It was not. The 94% statistic was an artifact of research methodology. Compton’s 173-subject sample consisted not of drivers on the road but of drivers already arrested for driving impaired by what non-DRE officers recognized to be drugs. This method skewed the study sample towards drug impairment; 94% had drugs on board. Once the sample group was selected, Compton’s DRE officers performed LEDA SA physical exams, after which they opined that every driver they tested had drug(s) present. These opinions were correct 94% of the time—because 94% of the sample group had drugs present. This methodology reveals not the diagnostic accuracy of the LEDA, but the prevalence of drugs in the sample population. On this sample had officers abandoned the LEDA and predicted “impairment” at random, for example by flipping a coin, they would have achieved the same 94% accuracy.^a^ Compton’s methodology failed to demonstrate that the LEDA-like tests the study investigated were any more accurate than random guessing.

The externally valid statistics commonly used to quantify the ability of a test to tell the difference between people who do and do not have the target condition are the paired statistics sensitivity and specificity. The question arises, “Can DIE sensitivity and specificity be extracted from Bigelow, Compton and Adler?”

Bigelow does allow calculation of the sensitivity of its non-reproducible LEDA-like testing for specific drug-dose combinations administered in the lab to healthy young men prescreened to pass the SFST. Because people who failed a screening SFST were excluded from enrollment in the study, Bigelow’s false positive count cannot be used to calculate specificity. (Bigelow’s section “Specificity” actually described PPV).

Compton and Adler cannot be used to calculate the sensitivity and specificity of the LEDA-like tests they studied, for two reasons.

First, the notion that there is one diagnostic accuracy describing how LEDA DRE Opinions identify *drugs* presumes “drug” is a homogenous medical entity. It is not. Different drugs affect people differently. The accuracy of DRE Opinions differs from drug to drug. Sensitivity for PCP is high. Sensitivity for benzodiazepines is low. There is no externally valid sensitivity for *PCP-and-benzodiazepines*. That number would vary as the prevalence of each drug varied and whatever the number was it would not correctly describe LEDA sensitivity for either PCP or benzodiazepine.

Second, considering each drug individually, Compton and Adler obtained chemical drug tests only when the driver failed the DIE. When the DRE Opinion (index) was “not impaired by a drug”—negative—blood and urine testing (reference) was not done (Adler included 26 exceptions to this rule). Because true negative and false negative results were excluded^b^, Compton and Adler’s study designs prevented them from collecting the information needed to calculate sensitivity and specificity.

Neither Bigelow, Compton nor Adler reported the externally valid statistics needed to calculate the diagnostic accuracy of the LEDA-like testing they investigated or of the DIEs now used by US law enforcement.

## Conclusion

Bigelow, Compton and Adler, the three validation studies commonly cited in American criminal prosecutions to quantify the accuracy of current US law enforcement DIE practice, did no reference testing of driving performance or physical or mental impairment, investigated tests different from those currently employed by US law enforcement, used methodologies that biased accuracies, and reported DIE accuracy statistics that are not externally valid. The LEDA accuracies reported by these studies do not quantify the accuracy of the DIE process now used by US law enforcement. These validation studies do not validate current DIE practice.

## Methods

### Defining question

This paper’s defining question has two parts. The first seeks to clarify current policy and practice: What scientific research is adduced by US law enforcement, and identified in criminal prosecutions, as evidence of the diagnostic accuracy of the police DIE process? The second part investigates whether the studies identified in part one do in fact validate current DIE practice: Do the methodologies used by these studies allow them to correctly quantify the diagnostic accuracy of Drug Influence Evaluations as currently administered by US law enforcement Drug Recognition Experts?

### Identifying sources

Publications of the DIE program-standardizing organization, the IACP, were reviewed, including Standards, the Drug Evaluation and Classification Program web site DECP.org, and the IACP-approved US law enforcement DRE Student Manual, which is widely used in American courts as a foundation for DIE testimony.

Personnel at the IACP were contacted. The Executive Director was contacted. An IACP committee, the Technical Advisory Panel (TAP), “provides the IACP Technical information and advice as requested concerning the DEC Program” [http://www.decp.org/oversight; retrieved March 4, 2013]. The IACP TAP chairman and the TAP medical expert were contacted. They kindly agreed to help. They were asked to identify what elements of current US law enforcement DIE testing are standardized, and what scientific research underlies those standards.

An official at the National College of DUI Defense (NCDD) was contacted. The NCDD is an organization of American criminal defense attorneys specializing in alcohol and drug impaired driving law. The NCDD is the only organization accredited by the American Bar Association to certify attorneys in the DUI Defense Law specialty practice area. A founding member and former Dean of the College, who is an attorney specializing in DUI law, a law professor and the author of a respected textbook of DUI law, was contacted and asked to identify the studies relevant to this paper’s defining question.

### Data extraction and evaluation

Studies were selected and their full reports were analyzed. Data were extracted and analyzed by a single reviewer. Extracted data included research methodologies pertinent to the 14 questions posed by the QUADAS tool. The analysis was done by matching the extracted methodologies with QUADAS items, seeking to identify whether the studies’ methodologies were likely to bias their reported accuracies. When a biasing methodology was identified, its likely effects on each study’s measurement of DIE diagnostic performance were considered. QUADAS presumes research quantifies diagnostic accuracy with statistics that are externally valid. The selected studies quantified DIE performance with accuracy statistics that are internally but not externally valid; the analysis considered the effects of this choice of statistics.

## Endnotes

^a^Of the 173 subjects considered in Compton’s accuracy calculation, a coin toss would on average predict drugs in 86.5. Of the 162 subjects with drugs on board, a coin toss would on average predict drugs in 81. Thus on average 81/86.5 = 94% of the coin’s “Yes, drugs” predictions would be correct.

^b^Compton and Adler did report what appear to be false negative counts. These exclude true false negatives (people falsely judged not drug impaired) and tally instead people with one drug on board who were wrongly judged to be impaired by some other drug. The faux sensitivities that may be calculated with these numbers represent only upper bounds of the actual sensitivities, the true numbers being unknown but certainly smaller. True negatives and specificity are similarly affected.

## Appendix 1

Validation studies’ research methodologies.

### Bigelow 1985

WHAT: Lab study.

NULL HYPOTHESIS: none. Self identified objective: “to provide information concerning the validity of subject examination procedures for identifying and differentiating types of drug intoxication” [Bigelow, page 1].

SUBJECT SELECTION: Young male volunteers were identified by newspaper advertisements, screened for good health, trained to do the SFST and excluded if they could not pass an SFST.

INDEX TEST: DRE officers did a physical exam and asked unstandardized questions. This procedure was explicitly different from the procedure used in the field. Officers formed a LEDA DRE Opinion as to whether the subject was impaired by a drug, and if so which category of drug. Available conclusions were limited to CNS stimulant, CNS depressant, marijuana, narcotic, or placebo. No subjects were given narcotics.

REFERENCE TEST: No reference testing was done. The reference “test” was the record of which dose of drug the person had been given. People given any dose of any drug were counted as impaired. People not given a drug were counted as not impaired.

ACCURACY REPORTED: Tables tallied officers’ predictions for each drug-dose combination. The study highlighted PPVs.

### Compton 1986

WHAT: Field study.

NULL HYPOTHESIS: none. Self identified objective: “To validate the LAPD [Los Angeles Police Department] drug recognition program”, by doing “a field study in which police officers employed the drug recognition procedure with real suspects under field conditions” [Compton, Abstract].

SUBJECT SELECTION: Drivers were stopped and assessed by non-DRE officers. Drivers judged not to be impaired were excluded. Drivers judged to be chemically impaired were arrested and breath alcohol levels were tested. Arrested drivers whom the non-DRE officers suspected of drug impairment were taken to the police station where they underwent a “[c]ursory examination to determine evidence of drug impairment” [Compton, page 34]. If drug impairment was not apparent on cursory exam, the driver was excluded. If drug impairment was apparent on cursory exam, a blood test for drugs was ordered. Drivers who refused drug testing were excluded. Remaining drivers underwent a LEDA SA examination, after which the DRE officer formed his LEDA Opinion as to whether the driver was drug impaired, and if so by which category of drug. Drivers who were judged not impaired were excluded. Subjects with alcohol levels above the legal limit were not excluded. Fifty-two percent of drivers in the final sample group had alcohol in their system, with an average BrAC of 0.06% mg/dl [Compton, page 14].

INDEX TEST: DRE officers’ LEDA Opinions.

REFERENCE TEST: Blood test for drugs. Any drug at any detectable level was interpreted as proving impairment. The principal metabolites of cocaine and marijuana were excluded; how other metabolites were tested and interpreted was not reproducibly described.

ACCURACY REPORTED: The final report highlighted the frequency with which officers’ predictions of a particular drug were associated with toxicology revealing any drug at any level: 94%. Contingency tables for some individual drugs were included (but see §Accuracies).

### Adler 1994

WHAT: Field study. Retrospective analysis of Phoenix, Arizona DRE records. Study results were reported at least four times [Adler, page 56]; this paper reviews the final report.

NULL HYPOTHESIS: none. Self identified objective: to discover whether “DRE methods accomplish their stated purpose, i.e., the correct identification of drug impairment, as demonstrated by DRE opinions and specimen analyses?” [Adler, page ix].

SUBJECT SELECTION: Drivers identified in Phoenix Police Department records as having been assessed by DRE officers, arrested for drugged driving, and having submitted to urine or blood testing between January 1989 and May 1994. Drivers judged not to be drug impaired were excluded (for unexplained reasons, twenty-six drivers judged not to be drug impaired were included). Subjects with alcohol in their system were not excluded; 19% had alcohol levels above the current per se legal limit of 0.05% mg/dl [Adler, page 54].

INDEX TEST: DRE officers’ LEDA Opinions.

REFERENCE TEST: Urine toxicology. Blood toxicology used occasionally, details not reproducibly described. Toxicology protocol was not reproducibly described. Testing of drug metabolites was not reproducibly described.

ACCURACY REPORTED: The paper highlighted the overall accuracy with which officers’ toxicology predictions matched at least one drug found by toxicology: 83.5% [Adler, page 34].

## Appendix 2

Results by study.

### Bigelow

The LEDA-like process investigated is not reproducible, was explicitly different from the LEDAs done at the time and identifiably different from LEDAs now relied on by US law enforcement. Reported accuracies were subject to selection bias, spectrum bias, misclassification bias, incorporation bias, and review bias. Highlighted accuracy statistics are not externally valid. Limited drug and dose specific sensitivities can be calculated, specificity and likelihood ratio cannot. Did demonstrate that LEDA-like physical exams have a low sensitivity.

### Compton

The LEDA-like process investigated is not reproducible. Reported accuracies were subject to selection bias, forensic selection bias, spectrum bias, misclassification bias, verification bias, differential verification bias, incorporation bias, and review bias. Highlighted accuracies statistics are not externally valid. LEDA sensitivity, specificity, and likelihood ratio cannot be calculated. Highlighted 94% “accuracy”, a methodological artifact of combining selection bias with PPV, fails to demonstrate that diagnostic accuracy is greater than a coin toss.

### Adler

The LEDA-like process investigated is not reproducible. Reported accuracies were subject to selection bias, forensic selection bias, spectrum bias, misclassification bias, verification bias, differential verification bias, incorporation bias, and review bias. Highlighted accuracies are not externally valid. LEDA sensitivity, specificity, and likelihood ratio cannot be calculated. Highlighted 83.5% “decisions supported by laboratory analysis” accuracy, calculated by discarding mistaken DRE Opinions, does not quantify either the overall accuracy or the PPV of individual DRE Opinions.

## Abbreviations

BrAC: Breath alcohol concentration; CNS: Central nervous system; DIE: Drug influence evaluation; DRE: Drug recognition expert; IACP: International association of chiefs of police; LEDA: Law enforcement drug assessment; LEDA SA: Law enforcement drug assessment, suspect assessment; NCDD: National college of DUI defense; NHTSA: National highway traffic safety administration; PCP: Phencyclidine; PPV: Positive predictive value; QUADAS: Quality assessment of diagnostic accuracy studies; SFST: Standardized field sobriety test.

## Competing interests

I’ve consulted on and given expert testimony about the diagnostic accuracy of traffic-police sobriety tests in a handful of criminal cases. I’ve done that free, or I’ve charged a nominal fee.
